# *KRAS*, *NRAS*, and *BRAF* mutation prevalence, clinicopathological association, and their application in a predictive model in Mexican patients with metastatic colorectal cancer: A retrospective cohort study

**DOI:** 10.1371/journal.pone.0235490

**Published:** 2020-07-06

**Authors:** Hector Eduardo Sanchez-Ibarra, Xianli Jiang, Elena Yareli Gallegos-Gonzalez, Adriana Carolina Cavazos-González, Yenho Chen, Faruck Morcos, Hugo Alberto Barrera-Saldaña

**Affiliations:** 1 Genetics Laboratory, Vitagénesis SA de CV, Monterrey, Nuevo Leon, Mexico; 2 Evolutionary Information Laboratory, Department of Biological Sciences, the University of Texas at Dallas, Richardson, Texas, United States of America; Ohio State University Wexner Medical Center, UNITED STATES

## Abstract

Mutations in *KRAS*, *NRAS*, and *BRAF* (*RAS/BRAF*) genes are the main predictive biomarkers for the response to anti-EGFR monoclonal antibodies (MAbs) targeted therapy in metastatic colorectal cancer (mCRC). This retrospective study aimed to report the mutational status prevalence of these genes, explore their possible associations with clinicopathological features, and build and validate a predictive model. To achieve these objectives, 500 mCRC Mexican patients were screened for clinically relevant mutations in *RAS/BRAF* genes. Fifty-two percent of these specimens harbored clinically relevant mutations in at least one screened gene. Among these, 86% had a mutation in *KRAS*, 7% in *NRAS*, 6% in *BRAF*, and 2% in both *NRAS* and *BRAF*. Only tumor location in the proximal colon exhibited a significant correlation with *KRAS* and *BRAF* mutational status (*p*-value = 0.0414 and 0.0065, respectively). Further t-SNE analyses were made to 191 specimens to reveal patterns among patients with clinical parameters and *KRAS* mutational status. Then, directed by the results from classical statistical tests and t-SNE analysis, neural network models utilized entity embeddings to learn patterns and build predictive models using a minimal number of trainable parameters. This study could be the first step in the prediction for *RAS/BRAF* mutational status from tumoral features and could lead the way to a more detailed and more diverse dataset that could benefit from machine learning methods.

## Introduction

Colorectal cancer (CRC) is the third most common cancer worldwide and its prevalence has increased due to external risk factors such as diet, obesity, and sedentary lifestyle [1]. In Mexico, cancer is the third most common cause of death and CRC is the fourth most frequent. From 2000 to 2012, the age-adjusted mortality rate per 100,000 inhabitants increased from 3.9 to 4.8 [2]. The most recent data from GLOBOCAN recorded 14,900 deaths from CRC in Mexico in 2018 [3]. The Mexican healthcare system is unable to cope with the increasing need for an early cancer diagnosis, a situation worsened by a poor preventive culture. As a result, almost 80% of CRC cases are diagnosed in advanced stages with a high probability of presenting metastasis leading to a poor prognosis [4].

Two of the available targeted treatments for metastatic CRC (mCRC) are based on monoclonal antibodies that inhibit the signaling pathway initiated by the epidermal growth factor binding to its receptor (EGFR). Mutations in genes that integrate the EGFR signaling cascade determine the response to this therapy, therefore they are used as predictive biomarkers. Previous studies have shown that *KRAS* (exons 2, 3, and 4), *NRAS* (exons 2, 3, and 4) and *BRAF* (codon 600 in exon 15) genes, all being part of the EGFR signaling cascade, are mutated in approximately 45–55% of the mCRC cases [5–8].

CRC is a very complex cancer that can be classified according to its pathological features. Many studies have found associations between these features or demographical data and genes mutational status showing interesting results. In a French cohort, mutations in the *KRAS* gene were more frequent in men, while an Australian study concluded that they were more frequent in women [9,10]. A study in Italy found an association between mucinous adenocarcinoma and *KRAS* mutations, but not with *NRAS* or *BRAF* mutations [11].

One of the approaches to find these associations, especially between the integration of multiple traits and the mutational status is through machine learning. In the last decade, machine learning has played a crucial role in effectively building data-driven biological models to predict cancer progression [12], susceptibility [13], recurrence [14], survival [15], and other clinical outcomes from complex datasets integrated by clinical and genomic features by discovering and identifying patterns and relationships among those features. Machine learning techniques used in cancer research include Artificial Neural Networks (ANNs) [16,17], Bayesian Networks (BNs) [18], and Support Vector Machines (SVMs) [19]. ANNs are powerful tools to study a broad range of cancers, including breast cancer [17], CRC [20], and lung cancer [21]. For example, the integration of mammographic and demographic data of breast cancer patients for ANN yields 96.5% of the accuracy of breast cancer risk prediction [17]. In this study, we used neural network model as our starting point for building a predictive model since its architecture allows it to learn high dimensional nonlinear data spaces, such as clinical datasets.

The aims of this study were to determine *KRAS*, *NRAS*, and *BRAF* mutation prevalence and their possible association between tumoral clinicopathological features in mCRC patients from Mexico. Also, these data were integrated as input features, including clinical variables and histological parameters, in machine learning algorithms to build and validate a model to predict and visualize the presence of mutations in the *KRAS* gene.

## Materials and methods

### Patients and biological specimens

Biospecimens for this retrospective study were acquired through the *KRAS*, *NRAS*, and *BRAF* mutation analysis service performed at the Genetics Laboratory at Vitagénesis S.A. de C.V. located in Monterrey, Mexico. The study was approved by the Ethics and Research Committee from Hospital La Misión S. A. de C. V. (17CI19039096) in the city of Monterrey, Mexico and all data were fully anonymized and the requirement for written informed consent was waived, given this study’s retrospective nature. This research was carried out following approved guidelines and the Declaration of Helsinki. Formalin-fixed paraffin-embedded (FFPE) primary and metastatic (only if the primary tumor was not available) tumor specimens from 546 mCRC patients from diverse hospitals across the country were retrospectively reviewed from January 2015 to June 2018. Specimens in which only *KRAS* was analyzed for mutations were excluded (n = 46), giving a total of 500 specimens for further analysis.

### Mutation screening

Five FFPE sections of 15 μm thickness were used per analysis. DNA was extracted with the QIAmp FFPE Tissue kit (Qiagen, Hilden, Germany) following manufacturer instructions. Mutation screening was performed for clinically relevant mutations in exons 2, 3, and 4 of *KRAS* and *NRAS*, as well as in codon 600 of exon 15 of *BRAF*. Specimens that arrived from 2015 to 2016 (n = 128) were analyzed only for *KRAS* and *NRAS* mutations by Sanger sequencing (Genetic Analyzer 3130, Applied Biosystems, Foster City, USA) [22]. From mid-2016 to 2018, specimens were evaluated using the fully-automated qPCR *KRAS* and *NRAS*3 (*NRAS* and *BRAF*) Idylla^™^ (Biocartis, Mechelen, Belgium) mutation assays (n = 370) [23,24]. From the latter specimens, those that did not meet Idylla^™^ requirements were screened for only *KRAS* and *NRAS* genes (n = 2) using the EntroGen RAS mutation screening panel (EntroGen, Tarzana, USA) in a StepOne^™^ Real-Time PCR System (Applied Biosystems, Foster City, USA), following manufacturer instructions.

### Histopathological features for statistical analyses

Histopathological information of these tumors was retrieved from paired reports by the institutions where the specimens came from. Those were High Specialty Regional Hospitals of Bajío (HRAEB), Oaxaca (HRAEO), Yucatan’s peninsula (HRAEPY), Chiapas (HRAEC), Ciudad Victoria (HRAECV), and Ixtapaluca (HRAEI), in addition to the following local clinics and hospitals: Doctor’s Hospital, Opción Oncología, ONCARE, OCA Hospital, Hospital San José, and Hospital Zambrano Hellion. All information was gathered and arranged into a database where it was normalized. The W.H.O. histological classification of tumors served as a guideline for the designation of the histological subtype classification (classical adenocarcinoma, mucinous adenocarcinoma, and signet-ring cell adenocarcinoma), histological grade (well, moderately, and poorly differentiated), and tumor site (proximal and distal colon, and rectum) [25]. Specimens located in cecum, ascending colon, and transverse colon were cataloged as proximal colon while those located in the descending colon and sigmoid colon as the distal colon. There was no available data on the specimens’ MSI status.

### Traditional statistical analyses

To identify possible associations between clinicopathological features and the mutational status, specimens were treated as two different groups: one used for the association analysis of *KRAS* and *NRAS* genes (n = 500) and a subset for the association analysis of *BRAF* gene (n = 370). Associations between mutational status and tumoral histopathological features were analyzed using a *χ*^*2*^-test for qualitative variables and opting for a Student’s *t*-test for quantitative variables (age only) [9]. The significance threshold was set at p <0.05. Traditional statistical analyses were performed using SPSS Windows (Version 16.0) software (SPSS Inc, Chicago, IL, USA).

### Data processing for machine learning

Ages were classified into 10-year range categories. Histological subtype data were categorized into four groups: classical adenocarcinoma, mucinous adenocarcinoma, signet-ring cell adenocarcinoma, and others. Cities of origin were grouped into five regions: West (or Bajío), Central, North, North Pacific Coast, and South, based on geography and dietary cultures (S1 Table) [26]. In general terms, Northern Mexico possesses a heavy meat diet with influences from American southwest food and Bajío people prefer deep-fried pork meat, while the North Pacific Coast consumes more vegetables, seafood, and fruits. Diet in the South is more indigenous and influenced by Mayan and Caribbean traditions. Central Mexico, which includes Mexico City, represents a mixture of dietary cultures not only from other Mexican regions but also from foreign countries. Tumor sites were grouped into eight potential categories based on location: colon, ascending colon, descending colon (including tumor sites at recto-sigmoid joint and sigmoid colon), hepatic system (gallbladder, liver, and hepatic node), rectum, small intestine (small intestine, ileum valve, duodenum, and cecum), transverse colon, and other (every specimen not included in other groups).

### t-distributed stochastic neighbor embedding (t-SNE)

In this study, we started by visualizing data of 190 patients using an unsupervised clustering and dimension reduction algorithm, t-SNE [27,28], to identify groups with similar multi-phenotypes-genotypes relationships. We utilized the t-SNE Scikit-Learn implementation in our dataset to evaluate the clinical characteristics pattern within gene mutation. Each subject is mapped from data space onto a single point in the 2D map space and was then color-coded according to their respective clinical characteristics. Clinical features are encoded in data space using a concatenated 1-of-k one-hot vector technique. All 2D map space embeddings reached a stable configuration within 2,000 iterations with a learning rate set to 300 and perplexity between 8 and 15. Patients with missing data were excluded. Absolute location and shape of t-SNE clusters typically have little real-world significance. However, clusters can be used to build intuition behind how certain features are linked together within the studied dataset and guide the construction of machine learning models.

### Neural networks

After excluding subjects with missing values in age, histological subtype, histological grade, tumor site, and city, the yielded sample size was 163 patients which were split into 90/10 train/test set, with 146/17 subjects, respectively. The prevalence of *KRAS* mutations in this subgroup is comparable with the whole group, having 41% and 45% of *KRAS* mutation rate respectively. We employed a 2-layer neural network using the Keras neural network library [29]. In this model, patients can be encoded as a 2D vector and those with similar traits will be mapped in proximity while patients with differing traits are mapped distally. We decided to see if a neural network model can learn to encode patients from tabular data onto 2D vector space and then use that vector as a way to classify whether they had *KRAS* mutations or not. In real practice, inputs were fed directly into an embedding layer, flattened, and passed to a two-neuron softmax output dense layer that predicted the presence of *KRAS* mutation against wild-type *KRAS*. The output of the model is the probability of each target class–contains *KRAS* mutation or does not contain *KRAS* mutation, and the class with the highest probability becomes the answer of the prediction (yes/no mutation). Categorical cross-entropy serves as the loss function for the backward propagation learning phase. In the 5-stratified randomized folds cross-validation method, the dataset was shuffled and split up into five non-overlapping sections where the *KRAS* mutation proportion remained equal to the training set proportion (41%). All models were trained using a 5-fold stratified shuffle split with results averaged across the tests. All networks reached a stable configuration within 200 epochs. ROC (Receiver Operating Characteristic) curves and validation prediction accuracy served as a model performance metric. Accuracy and loss curves were also reported to visualize the training cycle over each epoch. The accuracy is the proportion of correct predictions in the validation set. Since the model is trained over five different cross-validation folds, we average the prediction accuracy over all five folds.

## Results

### Mutation frequency

Fifty-two percent of the specimens were found to harbor clinically relevant mutations in *RAS/BRAF (KRAS*, *NRAS*, and *BRAF)*. Within these specimens (n = 263), 86% had a mutation in *KRAS*, 8% in *NRAS*, and 7% in *BRAF* (Table 1).

**Table 1:** Mutational status of patients with mCRC by genes and country regions.

	*KRAS* (n = 500, %)		*NRAS* (n = 500, %)		*BRAF* (n = 370, %)	
Country region	Wildtype	Mutated	Wildtype	Mutated	Wildtype	Mutated
North	150 (56%)	118 (44%)	254 (95%)	14 (5%)	198 (96%)	8 (4%)
North Pacific Coast	68 (60%)	46 (40%)	112 (98%)	2 (2%)	74 (93%)	6 (7%)
Bajío	25 (49%)	26 (51%)	50 (98%)	1 (2%)	35 (97%)	1 (3%)
South	19 (54%)	16 (46%)	34 (97%)	1 (3%)	23 (92%)	2 (8%)
Central	13 (41%)	19 (59%)	30 (94%)	2 (6%)	22 (96%)	1 (4%)
Total	275 (55%)	225 (45%)	480 (96%)	20 (4%)	352 (95%)	18 (5%)

Most of the specimens came from the north of Mexico (n = 268) and the least frequent region was Central (n = 33). This region had the highest *RAS/BRAF* mutational rate (69%) and the north had the lowest (51%).

Regarding the *KRAS* gene, 72% of the mutations were located in codon 12, 16% in codon 13, 6% in codon 146, 4% in codon 61, and 1% in codons 59 and 117. The most frequent mutation in *KRAS* was G12D composing 28% of mutations in the said gene. Mutations in *NRAS* were more common in codon 61 (prevalence of 55% in all the *NRAS* mutated specimens), while the rest were in codon 12, 13, and 117 (30%, 10%, and 5%, respectively) (Table 2). Two rare cases where the patients had mutations in both *NRAS* and *BRAF* were found, despite that these mutations are generally considered as mutually exclusive.

**Table 2:** Mutations by location and type in *RAS/BRAF* genes.

Gene Location	No. Mutated Cases per Gene		
Codon	*KRAS* (n = 225, %)	*NRAS* (n = 20, %)	*BRAF* (n = 18, %)
**Codon 12**	162 (72%)	6 (30%)	N/A
G12V	40 (18%)	0(0%)	N/A
G12D	73 (32%)	6 (30%)	N/A
G12C	17 (8%)	0(0%)	N/A
G12A	17 (8%)	0(0%)	N/A
G12S	11 (5%)	0(0%)	N/A
G12R	2 (1%)	0(0%)	N/A
G12V/D/A^†^	1 (0.4%)	0(0%)	N/A
G12C/S^†^	1 (0.4%)	0(0%)	N/A
**Codon 13**	36 (16%)	2 (10%)	N/A
G13A	36 (16%)	0(0%)	N/A
G13D	0(0%)	1 (5%)	N/A
G13R/V^†^	0(0%)	1 (5%)	N/A
**Codon 59**	2 (1%)	0(0%)	N/A
A59T/V^†^	2 (1%)	0(0%)	N/A
**Codon 61**	10 (4%)	11 (55%)	N/A
Q61H	2 (1%)	1 (5%)	N/A
Q61K	2 (1%)	3 (15%)	N/A
Q61L/R^†^	6 (3%)	7 (35%)	N/A
**Codon 117**	2 (1%)	1 (5%)	N/A
K117N/R/E^†^	2 (1%)	1 (5%)	N/A
**Codon 146**	13 (6%)	0(0%)	N/A
A146P/T/V^†^	13 (6%)	0(0%)	N/A
**Codon 600**	N/A	N/A	18 (100%)
V600E/D^†^	N/A	N/A	18 (100%)

*Presence of one of these mutations, not concomitant. N/A = Not applicable.

### General histopathological and clinical features

The average age of patients was 56.6 years and their distribution by sex was almost equal. From the specimens where tumor site information was available (n = 266), 44% of them were in the rectum, 21.4% were in the proximal colon, and 34.6% in the distal colon. The predominant histological subtype of the characterized specimens (n = 281) was classical adenocarcinoma with a prevalence of 86.1%, followed by mucinous carcinoma and signet ring cell carcinoma with an incidence of 9.6% and 2.1%, respectively. Histological grade was determined in 197 specimens, of which 12.7% were classified as well-differentiated, 72.6% as moderately differentiated, and 14.7% as poorly differentiated. Almost half of the tumors were reported to be in stage 4 (n = 236), and from 112 patients we determined that the liver is the most frequently affected organ by metastasis (n = 56) (Table 3).

**Table 3:** Associations between genetic and clinicopathological features.

	*KRAS*			*NRAS*			*BRAF*			Total
Variable	Mutated (n = 225, %)	Wild-type (n = 275, %)	p value	Mutated (n = 20, %)	Wild-type (n = 480, %)	p value	Mutated (n = 18, %)	Wild-type (n = 352, %)	p value	(n = 500, %)
**Age, Median**	57.4	56.1	0.9233	51.9	56.9	0.1629	63.4	56.6	0.0639	56.6
**Sex (n = 500)**			0.4662			1			0.814	
Female	107 (47.6%)	127 (46.2)		9 (45%)	225 (46.9%)		8 (44.4%)	168 (47.7%)		234 (46.8%)
Male	118 (52.4%)	148 (53.8%)		11 (55%)	255 (53.1%)		10 (55.6%)	184 (52.3%)		266 (53.2%)
**Tumor site (n = 266)**			0.0414*			0.2298			0.0065*	
Proximal colon	35 (29.2%)	22 (15.1%)		0 (0%)	57 (22.4%)		6 (46.2%)	38 (20.1%)		57 (21.4%)
Distal colon	35 (29.2%)	57 (39%)		4 (36.4%)	88 (34.5%)		7 (53.8%)	71 (37.6%)		92 (34.6%)
Rectum	50 (41.7%)	67 (45.9%)		7 (63.6%)	110 (43.1%)		0 (0%)	80 (42.3%)		117 (44%)
**Histological subtype (n = 281)**			0.1894			0.4984			0.9237	
Adenocarcinoma	102 (83.6%)	140 (88.1%)		14 (100%)	228 (85.4%)		9 (90%)	189 (85.1%)		242 (86.1%)
Mucinous carcinoma	16 (13.1%)	11 (6.9%)		0 (0%)	27 (10.1%)		1 (10%)	23 (10.4%)		27 (9.6%)
Signet ring cell carcinoma	1 (0.8%)	5 (3.1%)		0 (0%)	6 (2.2%)		0 (0%)	5 (2.3%)		6 (2.1%)
Others	3 (2.5%)	3 (1.9%)		0 (0%)	6 (2.2%)		0 (0%)	5 (2.3%)		6 (2.1%)
**Histological grade (n = 197)**			0.2758			0.7361			0.7684	
Well	4 (16.9%)	11 (9.6%)		2 (20%)	23 (12.3%)		2 (22.2%)	22 (13.8%)		25 (12.7%)
Moderate	56 (67.5%)	87 (76.3%)		7 (70%)	136 (72.7%)		6 (66.7%)	115 (71.9%)		143 (72.6%)
Poor	13 (15.7%)	16 (14%)		1 (10%)	28 (15%)		1 (11.1%)	23 (14.4%)		29 (14.7%)
**Clinical stage (n = 293)**			0.1538			0.1934			0.3535	
2	9 (7.1%)	4 (2.4%)		0 (0%)	13 (4.6%)		0 (0%)	10 (4.6%)		13 (4.4%)
3	18 (14.2%)	26 (15.7%)		0 (0%)	44 (15.7%)		3 (27.3%)	29 (13.3%)		44 (15%)
4	100 (78.7%)	136 (81.9%)		13 (100%)	223 (79.6%)		8 (72.7%)	179 (82.1%)		236 (80.6%)
**Metastasis site (n = 112)**			0.3794			0.9616			0.956	
Liver	22 (45.8%)	31 (48.4%)		2 (66.7%)	51 (46.8%)		1 (100%)	44 (47.8%)		53 (47.3%)
Lung	6 (12.5%)	5 (7.8%)		0 (0%)	11 (10.1%)		0 (0%)	8 (8.7%)		11 (9.8%)
Liver and lung	2 (4.2%)	3 (4.7%)		0 (0%)	5 (4.6%)		0 (0%)	5 (5.4%)		5 (4.5%)
Peritoneum	0 (0%)	5 (7.8%)		0 (0%)	5 (4.6%)		0 (0%)	4 (4.3%)		5 (4.5%)
Lymph node	16 (33.3%)	16 (25%)		1 (33.3%)	31 (28.4%)		0 (0%)	26 (28.3%)		32 (28.6%)
Ovary	2 (4.2%)	4 (6.3%)		0 (0%)	6 (5.5%)		0 (0%)	5 (5.4%)		6 (5.4%)

*Significance threshold at p <0.05.

### Traditional statistical associations between *RAS/BRAF* mutational status and patients´ features

*KRAS* mutation screening revealed that 45% (n = 225) of the specimens harbored a mutation against 55% of *KRAS*-wildtype specimens. No significant difference in age was found, *KRAS*-mutants had an average of 57.4 years and *KRAS*-wildtypes an average of 56.1. In terms of sex, no significant difference was found between male and female patients. Tumors located in the proximal colon were found to be significantly associated with the presence of mutations in the *KRAS* gene in contrast to wild-type specimens (29.2% vs. 15.1%, *p-value* = 0.0414). *KRAS*-mutated and tumor histological classification (classical adenocarcinoma, 83.6%), the histological grade of differentiation (moderate differentiation, 67.5%), and clinical stage (stage IV, 78.8%) display an apparent tendency; however, there was no statistically significant difference to support these premises (Table 3). Furthermore, we probed the possible association between mutations among exons and codons, but no significant difference was found, either (S1 and S2 Tables).

Regarding *NRAS* analysis, 4% were *NRAS*-mutants. No association was found between the mutational status of *NRAS* and any clinicopathological feature. All *NRAS*-mutated specimens were in the distal colon or rectum. The rectum was the most common *NRAS-*mutated site (63.6% vs. 43.1%) and there was a tendency in specimens with this genotype for well-differentiated tumors (20% vs. 12.3%) but with no definitive statistically significant difference.

Analysis of the *BRAF* gene revealed that 5% (n = 18) were mutated. *BRAF*-mutated patients were older than *BRAF*-wildtypes (63.4 and 56.6, respectively), however, no significant difference was found (*p*-value = 0.0639). Also, a tendency was found in the histological subtype, with a bias towards classical adenocarcinoma (90% vs. 85.1%) although not statistically significant. The only association found with *BRAF*-mutated was the tumor location in the proximal colon (46.2% vs. 20.1% *p*-value 0.0065) as shown in Table 3.

### t-SNE clustering of *KRAS* mutational status with integrated patients’ features

**Fig 1:**
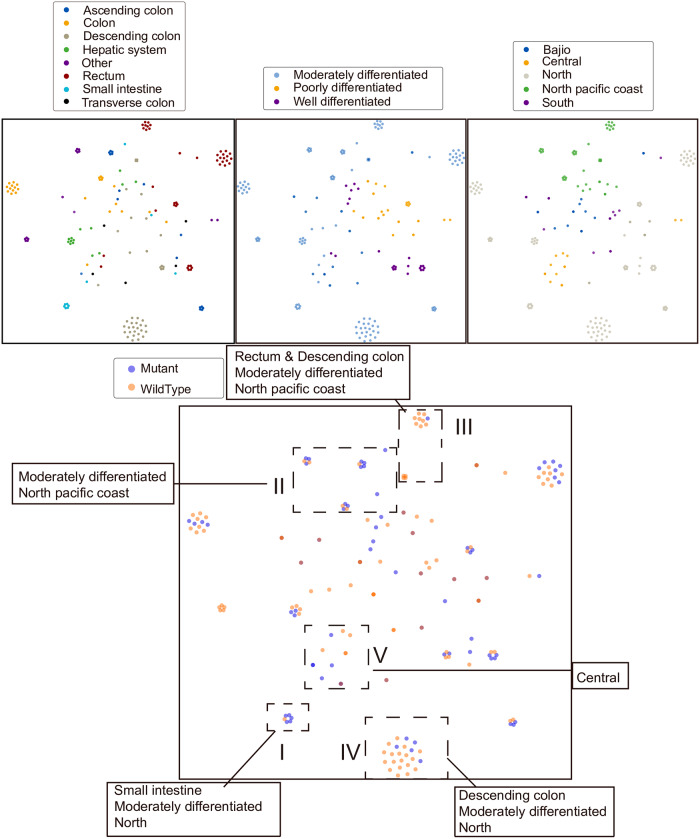
t-SNE clustering based on 3 clinical features (tumor site, histological grade, and city). And blindly colored *KRAS* mutation. Five selected clusters were annotated. Each data point represents a patient with a specific color indicating the subgroup of a clinical feature.

To further research the association of integrated features including more than one clinical and histopathological traits and the *KRAS* mutational status, t-SNE was used to analyze the data. It captures local topological patterns in high dimensional spaces and clustering them together on a low dimensional space. For our dataset, the best clusters were formed using three input features–histological grade, tumor site, and city–with genetic *KRAS* characteristics blindly color labeled to reveal interesting patterns and separation. City information was grouped according to the physical location and dietary traditions in mind (S1 Table). Tumor site information, which was found to be significantly associated with *KRAS* mutation (Table 3), was broadly grouped into different main organ loacation. Clustering errors may be introduced as some patients had tumor sites labeled under ‘Colon’, while others had a more specified tumor site labeling (i.e. Ascending, Descending, or Transverse Colon). Additionally, we also integrated the histological grade of differentiation, which was found associated, but not statistically significant to *KRAS*. Age and sex information were intentionally excluded since χ2 tests indicating that these features are poor predictors for the *KRAS* genotype (Table 3). The histological subtype and clinical-stage features were also excluded due to insufficiently large subcategories such as signet ring carcinoma histological type and clinical stage 2 having less than ten cases for *KRAS* mutants and wildtypes. Overall, 190 patients possessed complete sets of data and were included in the clustering algorithm.

t-SNE clustering allowed us to easily visualize multiple clinical features in comparison at once (Fig 1). Five clusters were annotated in the blindly labeled plot (lower panel of Fig 1) by using the t-SNE maps trained on the information from the three smaller subplots (upper panel of Fig 1). Cluster I shows that patients from Northern Mexico with tumors located in the small intestine and moderately differentiated histological grade exhibited a strong preference for *KRAS* mutation. Cluster II shows that patients from the North Pacific Region generally showed a high preference for *KRAS* mutation while cluster III suggests that *KRAS* mutations were unfavorable in the lower digestive tract, namely the descending colon and rectum which display a strong preference for wild type *KRAS* mutation. Cluster IV and V, representing North and Central Mexico patients, respectively, show that patients with tumor sites located in the descending colon and rectum also heavily favor the *KRAS* wild type status, suggesting that rectum and descending colon tumor sites preference for wild type *KRAS* status may be independent of diet and location.

### Predictive models for *KRAS* mutations by neural network

**Fig 2:**
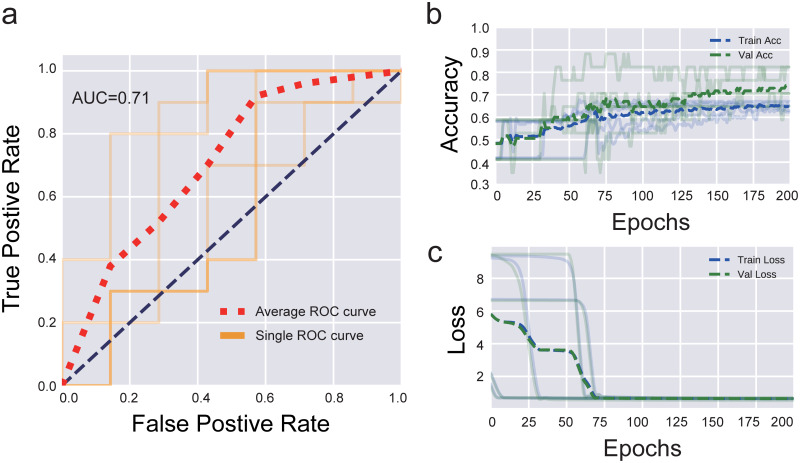
Performance of the neural network model by using continuous age a) ROC curve of the predictive model for *KRAS* mutation by using continuous age, histological subtype, histological grade, tumor site, and city. Accuracy (b) and loss (c) curves for both training and validation datasets during model training converge to a more stable configuration.

Neural network predictive modeling was constructed using the same input features used in t-SNE clinical characteristic plus histological type clustering, which exhibited an apparent tendency not statistically significant (Table 3), and with *KRAS* as the predicting feature since it has relatively even number of mutants and wild type cases, 45% and 55%, respectively. *BRAF* and *NRAS* gene information were excluded to reduce signal noise since these features are dependent on the *KRAS* genotype [30]. The ROC curve (S1 Fig) shows that the predictive model built with the above four features outperformed a random classifier (with AUC at 0.7) and indicates that these features may potentially hold valuable information connecting the *KRAS* mutation status and patient’s clinical and histopathological characteristics in a combinatorial manner. The model achieves accuracy far above chance with good statistical reliability (p<0.001) [31] including a 65.9% average validation accuracy (S1b and S1c Fig). Improvements to the predictive model were accomplished when using age as a continuous variable as opposed to the 10-year categories previously used (Fig 2). Not only does the ROC curve show slight improvement with an AUC with 0.71 (Fig 2a), the accuracy and loss curves during model training converge to a more stable configuration with less variation and a more consistent increase in validation accuracy across the 5-fold shuffle split (Fig 2b and 2c). This model achieves a far superior accuracy in contrast to the one before with statistical reliability p<0.001 and an average validation accuracy of 74.1%.

## Discussion

### Distribution and demographics

A pharmacoeconomic study revealed that only 63% of Latin American oncologists resort to genetic testing for *KRAS* (*NRAS* and *BRAF* not regularly included in the test) after an mCRC diagnosis [32]. This figure implies that a significant amount of mCRC patients undergo through a mismanaged therapy. The first molecular epidemiology study of CRC to be carried out in the Mexican population stated that 38% of the analyzed specimens contained mutations in the *KRAS* gene (only codons 12, 13, and 61 of *KRAS* were screened) [33]. A second study carried out in our laboratory reported a frequency of 35% of mutated specimens only in exon 2 of *KRAS* gene, albeit in this study where exon 3 and 4 of this gene, and both *NRAS* and *BRAF* genes were added to the analysis (the first of this scope in Mexico and Latin America), the frequency increased to 45% [22]. Therefore, it is necessary to adopt extended *RAS+BRAF* testing as a routine diagnostic procedure for a more precise approach.

A multicenter study conducted in several countries of Europe, Asia, and Latin America in 2010 reported that Hispanic CRC patients have the highest mutation rate in *KRAS* exon 2 (36% for European patients, 22% for Asian patients, and 40% for Hispanic patients) [32]. Other studies in Latin America report contrasting frequencies ranging from 13% (Colombia and Venezuela) to 40% (Chile). However, the literature shows that the *RAS* mutation prevalence usually has a range of 26% to 50% [34–36].

Previous studies have shown that mutations in *KRAS* are variable among populations. Studies in Spanish, Slovenian, Greek, Turkish, and Serbian populations reported *KRAS* mutation frequencies of 48%, 46.2%, 29.3%, 33.2%, and 35%, respectively, while Middle Eastern population reported frequencies of 48% (Iraq) and 32% (Saudi Arabia). The only study in an African population, conducted in Morocco, registered a *KRAS* mutation frequency of 23.9% [37]. Studies performed in Asian populations report *KRAS* mutation prevalence varying from 37.9% (Japan) to 52.7% (China), whereas *NRAS* and *BRAF* mutations prevalence are closer with China reporting 3.4% and 4.5% while Japan referring 4.2% and 5.4%, respectively [8,38], nevertheless, population-based studies report up to 20% of CRC patients with *BRAF* mutations [39,40]. This difference could be due since every specimen was screened for *KRAS*, but only those that were *KRAS* wild type were screened for *BRAF* since *KRAS* and *BRAF* mutations are generally mutually exclusive. There is a lack of studies in countries neighboring south of Mexico (Central America), however, a study performed in the north of it, in the United States, reported similar results [41].

### Traditional statistical associations

Using the dataset described in Table 3, the χ^2^ analyses yielded a statistically significant association between *KRAS* mutational status and tumors located in the proximal colon (*p*-value = 0.0414. However, *BRAF* mutational status was found to be more significantly associated (*p*-value = 0.0065) with proximal colon located tumors. These two associations could be explained by the fact that only *KRAS* wild-type specimens were screened for *BRAF*. These results coincide with the association between *BRAF* (but no *KRAS*) mutational status and the proximal colon in Chinese, Australian, and Swedish populations [8,10,40].

Nevertheless, several associations previously reported could not be replicated in our study. A tendency was found in the Japanese population where mutations in *KRAS* or *NRAS* were more common in rectal tumors, while our analysis showed a tendency in the proximal colon for *KRAS*-mutated and rectal tumors for *NRAS*-mutated [38]. In China, an association was found in *KRAS* exon 2 with older patients, whereas we found a non-significant tendency between *BRAF* and age. Also, we could not identify an association either with histologic subtype or grade as these studies did.

A large study in French CRC patients reported that *KRAS* mutants, *KRAS* exon 2 mutants, and *KRAS* codon 12 mutants were associated with the same clinicopathological features: male sex, classical adenocarcinoma, and well/moderately differentiated histological grade. Also, *KRAS* mutations in exons 3 and 4 were associated with rare histological subtypes [9]. We could not find an association of this type due to the lack of rare histological subtypes in our samples. The association between *BRAF* and tumor location found in our analysis was not found in the French study.

A study in the United States reported that *NRAS* mutations were associated with rectal tumors and *BRAF* mutations to old age [41] in agreement with our findings. Arguably, diet and environment could have a more significant role than the genetic load on the frequency of CRC in Mexican patients, more remarkably nowadays, when part of the diet and lifestyle of Mexico and the US are becoming more similar through globalization. Several studies have reported an association between a Western diet with a higher risk of CRC [42–44].

### t-SNE associations and neural network predictive model

t-SNE is an unsupervised clustering algorithm that has been shown to effectively map local nonlinear structures from high dimensional data space onto a lower-dimensional embedded map space. t-SNE clusters can be used to build intuition behind how certain features are linked together within our dataset and guide the focus of future clinical studies. To explore the associations between multiple clinical features and *KRAS* mutations, we have employed t-SNE to find the mutational patterns in a particular subgroup sharing three common clinical and histological features, including city, tumor site, histological grade, and then demonstrated that some clinical and histological features hold strong predictive properties for genetic information supported by the validation accuracy at 74.1% in artificial neural network models. In the ANNs models, we explored the use of entity embeddings, traditionally employed in natural language processing to represent sparse categorical data. Entity embeddings can be implemented as part of a supervised dense neural network and allow for learning of an optimal 2D map space where similar features are theoretically mapped close together [41]. As a result, we show that given an appropriate subset of input features instead of single features, it is possible to distinguish between individuals with *KRAS* mutation and those without it with high accuracies, that works well above chance accuracy using a minimal number of trainable parameters. Thus, we have established the potential of identifying several clinical and histopathological features as strong features relevant to mutational status.

Combining the weak pairwise associations reflected in our initial statistical analysis (age, histological subtype, histological grade, and, region) in machine learning models fuses the separate, typically weak, associations between each of the features with mutational status and therefore strengthens the ability to discern mutation preference in a certain group with same clinical patterns like histological subtype, histological grade, region, and, tumor location. Larger datasets routinely gathered immediately after or during diagnosis, and with larger sample size and including more clinical features could help validate further these associations between mutation and clinical traits for both neural network and t-SNE clustering method. Machine learning algorithms are only as good as the input data. More and cleaner data would help capture general population variances and minute patterns that we currently do not have enough information to accurately model. Since this model was optimized for Mexican patients, this particular model architecture has been proved to perform well on a small dataset, we expect a larger dataset to generate state-of-the-art performance. The neural network model has been widely used in pharmacogenetics to help predict drug efficacy in patients. Also, the interactions between genetic and clinical features modeled by neural networks may be eventually translated into individualized therapy at the clinical level. Therefore, more comprehensive data need to be collected in later study.

### Model and analysis limitations

Since our study population was chosen from a larger patient group based on their clinical data available is possibly subject to selection bias. These data should be interpreted with precaution in mind. Regarding our statistical analyses, several factors could affect the results, such as the technology used in each study and the number of specimens. Results reported in the Asian population are the most similar to those reported in this article given their comparable range of samples (n = 400–500) [8,38]. However, the study in the French population that managed to find associations with almost all the clinicopathological features had a total of 1793 samples [9]. Within our data, we could not find a significant difference in tumor location in *NRAS*-mutated samples even if all specimens came from distal colon or rectum tumors; likewise, in *BRAF* mutational status there was no significant difference between the age of the patients and the presence of gene mutations (*p*-value = 0.06). Therefore, it should be noted that these and even more associations could be found using larger sample sizes.

The same applies to our computational models. Although t-SNE showed promising blind cluster formation, its embedding algorithm is suboptimal for our specific dataset where features are unbalanced and there is reduced availability from the complete dataset (<500 patients). Larger datasets could help to further validate these associations between mutation and clinical features for both neural networks and the t-SNE clustering method. With a larger sample size, features from this dataset can be further combined with other quantitative biological features (proteomic data, cancer imaging data, more gene data, etc.) to build more complex models that can potentially anticipate treatment prediction, disease prognosis, and recurrence risk in CRC patients.

## Conclusions

These methodologies successfully extracted informative features contributing to *KRAS* mutation prediction and the underlying dependency of the features to indirectly estimate the associations between genes and clinical traits. This is the first study of this magnitude in Latin America. Approximately half of the Mexican patients with mCRC have a mutation that renders anti-EGFR treatment ineffective. Patients whose tumor is in the proximal colon are more likely to harbor mutations in *KRAS* or *BRAF*. t-SNE and Artificial Neural Network analyses showed systematic associations between tumor location, age, city of origin, histological subtype, and histological grade and *KRAS* mutational status. A predictive model built on these features shows the capacity to discern between patients with and without clinically relevant mutations in *KRAS* with an accuracy of 74.1% and motivates to spark a collection of larger datasets. Further analyses increasing the number of patients should improve the robustness and accuracy of our predictive model.

## Supporting information

S1 Fig Performance of the neural network model by using age in categories. a) ROC curve of the predictive model for *KRAS* mutation by using categorized age, histological subtype, histological grade, tumor site, and city. Accuracy (b) and loss (c) curves for both training and validation datasets during model training show wide variation with different among different folds.
(DOCX)

S1 Table City classification strategy. (DOCX)

S2 Table Association between *KRAS*-mutated exons and clinicopathological features. (DOCX)

S3 Table Association between *KRAS*-mutated codons and clinicopathological features. (DOCX)
