# Supplementary material for: KRAS, NRAS, and BRAF mutation prevalence, clinicopathological association, and their application in a predictive model in Mexican patients with metastatic colorectal cancer: A retrospective cohort study
Source: PLoS One. 2020 Jul 6;15(7):e0235490. doi: 10.1371/journal.pone.0235490 (PMC7337295; doi:10.1371/journal.pone.0235490)
Supplement: S1 Fig — a) ROC curve of the predictive model for KRAS mutation by using categorized age, histological subtype, histological grade, tumor site, and city. Accuracy (b) and loss (c) curves for both training and validation datasets during model training show wide variation with different among different folds. (DOCX) [file pone.0235490.s001.docx]

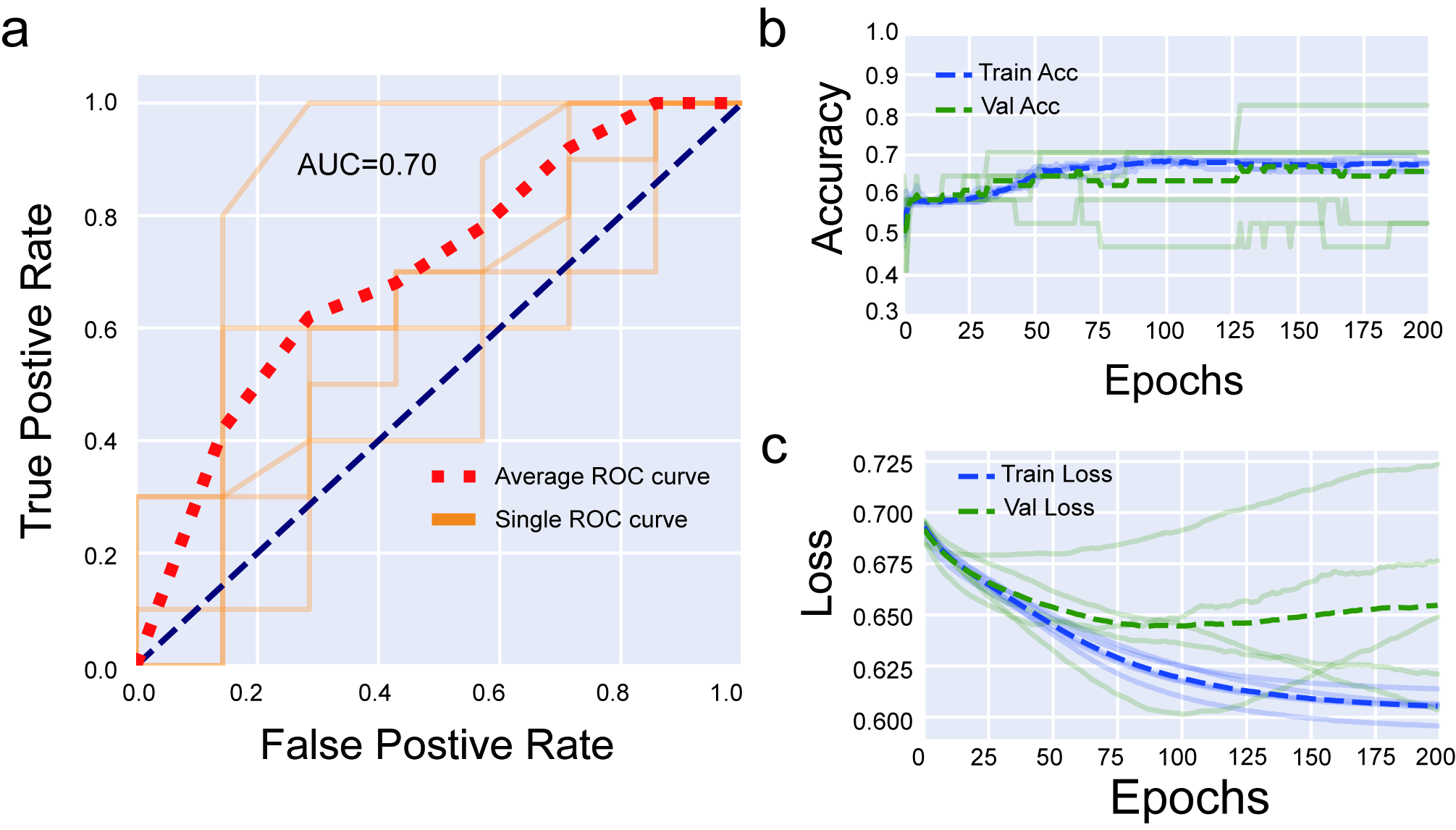
 **S1 Fig** Performance of neural network model by using age in categories a) ROC curve of the predictive model for *KRAS* mutation by using categorized age, histological subtype, histological grade, tumor site, and city. Accuracy (b) and loss (c) curves for both training and validation datasets during model training show wide variation with different among different folds.
