# Supplementary material for: KRAS, NRAS, and BRAF mutation prevalence, clinicopathological association, and their application in a predictive model in Mexican patients with metastatic colorectal cancer: A retrospective cohort study
Source: PLoS One. 2020 Jul 6;15(7):e0235490. doi: 10.1371/journal.pone.0235490 (PMC7337295; doi:10.1371/journal.pone.0235490)
Supplement: S1 Table — (DOCX) [file pone.0235490.s002.docx]

**S1 Table.** City classification strategy.

| North | North Pacific Coast | Bajío | South | Central |
| --- | --- | --- | --- | --- |
| Baja California Sur  Chihuahua  Nuevo León  Durango  Zacatecas  Tamaulipas  Aguascalientes  Coahuila de Zaragoza | Sonora  Jalisco  Colima  Nayarit  Sinaloa | San Luis Potosí  Guanajuato  Michoacán de Ocampo  Queretaro | Quintana Roo  Campeche  Yucatan | Ciudad de México  Mexico  Puebla  Hidalgo  Veracruz de Ignacio de la Llave |
