# Supplementary material for: KRAS, NRAS, and BRAF mutation prevalence, clinicopathological association, and their application in a predictive model in Mexican patients with metastatic colorectal cancer: A retrospective cohort study
Source: PLoS One. 2020 Jul 6;15(7):e0235490. doi: 10.1371/journal.pone.0235490 (PMC7337295; doi:10.1371/journal.pone.0235490)
Supplement: S2 Table — (DOCX) [file pone.0235490.s003.docx]

**S2 Table**. Association between *KRAS*-mutated exons and clinicopathological features.

|  | ***KRAS*** | | |  |  |
| --- | --- | --- | --- | --- | --- |
| Variable | **Exon 2** (n=198, %) | **Exon 3**  (n=12, %) | **Exon 4**  (n=15, %) | **p value** | **Mutated *KRAS***  (n=225) |
| **Age, Median** | 57.2 | 55.1 | 61.8 | 0.4 | 57.4 |
| **Gender (n=225)** |  |  |  |  |  |
| Female | 96 (48.5%) | 6 (50%) | 5 (33.3%) | 0.5 | 107 |
| Male | 102 (51.5%) | 6 (50%) | 10 (66.7%) |  | 118 |
| **Tumor site (n=120)** |  |  |  |  |  |
| Proximal Colon | 32 (30.5%) | 1 (12.5%) | 2 (28.6%) | 0.7 | 35 |
| Distal Colon | 29 (27.6%) | 4 (50%) | 2 (28.6%) |  | 35 |
| Rectum | 44 (41.9%) | 3 (37.5%) | 3 (42.9%) |  | 50 |
| **Histologic subtype (n=122)** |  |  |  |  |  |
| Adenocarcinoma | 90 (84.1%) | 6 (100%) | 6 (66.7%) | 0.7 | 102 |
| Mucinous carcinoma | 13 (12.1%) | 0 (0%) | 3 (33.3%) |  | 16 |
| Signet ring cell carcinoma | 1 (0.9%) | 0 (0%) | 0 (0%) |  | 1 |
| Others | 3 (2.8%) | 0 (0%) | 0 (0%) |  | 3 |
| **Histologic grade (n=83)** |  |  |  |  |  |
| Well | 13 (17.1%) | 1 (25%) | 0 (0%) | 0.8 | 14 |
| Moderate | 51 (67.1%) | 3 (75%) | 2 (66.7%) |  | 56 |
| Poor | 12 (15.8%) | 0 (0%) | 1 (33.3%) |  | 13 |
| **Clinical stage (n=127)** |  |  |  |  |  |
| 2 | 8 (7.5%) | 1 (9.1%) | 0 (0%) | 0.8 | 9 |
| 3 | 15 (14%) | 2 (18.2%) | 1 (11.1%) |  | 18 |
| 4 | 84 (78.5%) | 8 (72.7%) | 8 (88.9%) |  | 100 |
| **Metastasis site (n=48)** |  |  |  |  |  |
| Liver | 20 (47.6%) | 1 (50%) | 1 (25%) | 0.6 | 22 |
| Lung | 6 (14.3%) | 0 (0%) | 0 (0%) |  | 6 |
| Liver and lung | 2 (4.8%) | 0 (0%) | 0 (0%) |  | 2 |
| Peritoneum | 0 (0%) | 0 (0%) | 0 (0%) |  | 0 |
| Lymph node | 13 (31%) | 1 (50%) | 2 (50%) |  | 16 |
| Ovary | 1 (2.4%) | 0 (0%) | 1 (25%) |  | 2 |
| *Significance threshold at p <0.05. | |  |  |  |  |
