# Supplementary material for: KRAS, NRAS, and BRAF mutation prevalence, clinicopathological association, and their application in a predictive model in Mexican patients with metastatic colorectal cancer: A retrospective cohort study
Source: PLoS One. 2020 Jul 6;15(7):e0235490. doi: 10.1371/journal.pone.0235490 (PMC7337295; doi:10.1371/journal.pone.0235490)
Supplement: S3 Table — (DOCX) [file pone.0235490.s004.docx]

**S3 Table.** Association between *KRAS*-mutated codons and clinicopathological features.

|  | ***KRAS*** | | | | | |  | |  |
| --- | --- | --- | --- | --- | --- | --- | --- | --- | --- |
| Variable | **Codon 12**  (n=162, %) | **Codon 13**  (n=36, %) | **Codon 59**  (n=2, %) | **Codon 61**  (n=10, %) | **Codon 117**  (n=2, %) | **Codon 146**  (n=13, %) | | **p value** | ***KRAS* Mutated**  (n=225) |
| **Age, Median** | 57.1 | 57.5 | 51.5 | 56.1 | 71.0 | 60.0 | | 0.7 | 57.4 |
| Gender (n=225) |  |  |  |  |  |  | |  |  |
| Female | 81 (50%) | 15 (41.7%) | 2 (100%) | 4 (40%) | 1 (50%) | 4 (30.8%) | | 0.57 | 107 |
| Male | 81 (50%) | 21 (58.3%) | 0 | 6 (60%) | 1 (50%) | 9 (69.2%) | |  | 118 |
| **Tumor site (n=120)** |  |  |  |  |  |  | |  |  |
| Proximal Colon | 27 (31%) | 5 (27.8%) | 0 (0%) | 1 (14.3%) | 0 (0%) | 2 (33.3%) | | 0.95 | 35 |
| Distal Colon | 25 (28.7%) | 4 (22.2%) | 0 (0%) | 4 (57.1%) | 0 (0%) | 2 (33.3%) | |  | 35 |
| Rectum | 35 (40.2%) | 9 (50%) | 1 (100%) | 2 (28.6%) | 1 (100%) | 2 (33.3%) | |  | 50 |
| **Histologic subtype (n=122)** |  |  |  |  |  |  | |  |  |
| Adenocarcinoma | 76 (86.4%) | 14 (73.7%) | 0 (0%) | 6 (100%) | 1 (100%) | 5 (62.5%) | | 0.3 | 102 |
| Mucinous carcinoma | 8 () | 5 (26.3%) | 0 (0%) | 0 (0%) | 0 (0%) | 3 (37.5%) | |  | 16 |
| Signet ring cell carcinoma | 1 (%) | 0 (0%) | 0 (0%) | 0 (0%) | 0 (0%) | 0 (0%) | |  | 1 |
| Others | 3 (3.4%) | 0 (0%) | 0 (0%) | 0 (0%) | 0 (0%) | 0 (0%) | |  | 3 |
| **Histologic grade (n=83)** |  |  |  |  |  |  | |  |  |
| Well | 8 (12.9%) | 5 (35.7%) | 0 (0%) | 1 (25%) | 0 (0%) | 0 (0%) | | 0.6 | 14 |
| Moderate | 44 (71%) | 7 (50%) | 0 (0%) | 3 (75%) | 0 (0%) | 2 (66.7%) | |  | 56 |
| Poor | 10 (16.1%) | 2 (14.3%) | 0 (0%) | 0 (0%) | 0 (0%) | 1 (33.3%) | |  | 13 |
| **Clinical stage (n=127)** |  |  |  |  |  |  | |  |  |
| 2 | 7 (8%) | 1 (5%) | 0 (0%) | 1 (11%) | 0 (0%) | 0 (0%) | | 0.7 | 9 |
| 3 | 11 (13%) | 4 (21%) | 0 (0%) | 2 (22%) | 1 (50%) | 0 (0%) | |  | 18 |
| 4 | 70 (79.5%) | 14 (73.7%) | 2 (100%) | 6 (66.7%) | 1 (50%) | 7 (100%) | |  | 100 |
| **Metastasis site (n=48)** |  |  |  |  |  |  | |  |  |
| Liver | 16 (47.1%) | 4 (50%) | 0 (0%) | 1 (50%) | 0 (0%) | 1 (33.3%) | | 0.9 | 22 |
| Lung | 6 (17.6%) | 0 (0%) | 0 (0%) | 0 (0%) | 0 (0%) | 0 (0%) | |  | 6 |
| Liver and lung | 2 (5.9%) | 0 (0%) | 0 (0%) | 0 (0%) | 0 (0%) | 0 (0%) | |  | 2 |
| Peritoneum | 0 (0%) | 0 (0%) | 0 (0%) | 0 (0%) | 0 (0%) | 0 (0%) | |  | 0 |
| Lymph node | 9 (26.5%) | 4 (50%) | 0 (0%) | 1 (50%) | 1 (100%) | 1 (33.3%) | |  | 16 |
| Ovary | 1 (2.9%) | 0 (0%) | 0 (0%) | 0 (0%) | 0 (0%) | 1 (33.3%) | |  | 2 |
| *Significance threshold at p<0.05. | |  |  |  |  |  | |  |  |
